# Increased NTPDase Activity in Lymphocytes during Experimental Sepsis

**DOI:** 10.1100/2012/941906

**Published:** 2012-05-03

**Authors:** Claudia de Mello Bertoncheli, Carine Eloise Prestes Zimmermann, Jeandre Augusto dos Santos Jaques, Cláudio Alberto Martins Leal, Jader Betsch Ruchel, Bruna Cipolatto Rocha, Kelly de Vargas Pinheiro, Viviane do Carmo Gonçalves Souza, Daniel Roulim Stainki, Sônia Cristina Almeida Luz, Maria Rosa Chitolina Schetinger, Daniela Bitencourt Rosa Leal

**Affiliations:** ^1^Centro de Ciências da Saúde, Departamento de Microbiologia e Parasitologia, Universidade Federal de Santa Maria, 97105-900 Santa Maria, RS, Brazil; ^2^Centro de Ciências da Saúde, Departamento de Patologia, Universidade Federal de Santa Maria, 97105-900 Santa Maria, RS, Brazil; ^3^Centro de Ciências Naturais e Exatas, Departamento de Química, Universidade Federal de Santa Maria, 97105-900 Santa Maria, RS, Brazil

## Abstract

We investigated in rats induced to sepsis the activity of ectonucleoside triphosphate diphosphohydrolase (NTPDase; CD39; E.C. 3.6.1.5), an enzyme involved in the modulation of immune responses. After 12 hours of surgery, lymphocytes were isolated from blood and NTPDase activity was determined. It was also performed the histology of kidney, liver, and lung. The results demonstrated an increase in the hydrolysis of adenosine-5′-triphosphate (ATP) (*P* < 0.01), but no changes regarding adenosine-5′-monophosphate (ADP) hydrolysis (*P* > 0.05). Histological analysis showed several morphological changes in the septic group, such as vascular congestion, necrosis, and infiltration of mononuclear cells. It is known that the intracellular milieu contains much more ATP nucleotides than the extracellular. In this context, the increased ATPasic activity was probably induced as a dynamic response to clean up the elevated ATP levels resulting from cellular death.

## 1. Introduction

Sepsis is characterized by an inflammatory reaction as a consequence of immune system response to bacterial infection [1, 2]. The immune system has an important role in the pathogenesis of sepsis, which may cause tissue damage and lead to organic failure [3, 4]. The main process involves the activation of inflammatory cells such as leukocytes, tissue macrophages, dendritic cells, and eosinophils [5]. The exacerbated activation of innate immune response is one of the main components involved in the physiopathology of sepsis, which can be identified by increased proinflammatory factors after infection [6].

The membrane bound enzyme ectonucleoside triphosphate diphosphohydrolase (NTPDase; CD39; E.C. 3.6.1.5) modulates adenine nucleotides level, which are fundamental to the modulation of immune responses [7]. The enzymes of this family are widely distributed in animal tissues and represent the main ectoenzyme expressed by endothelial cells and muscle cells of the circulatory system [8, 9]. Under physiological conditions, the nucleotides are present in the extracellular environment in low concentrations, usually nanomolar, but may be found up to micromolar levels [10]. It is known that extracellular ATP, for example, when in micromolar concentrations, can induce the formation of pores in the cell membranes, resulting in osmotic changes [11], and it can also induce two antagonistic effects: cell proliferation, when in low concentrations, and cell death, when in high concentrations [10].

Considering the involvement of adenine nucleotides hydrolysis in the modulation of immune system and the participation of immune response in sepsis, the purpose of this study was to evaluate the hydrolysis of ATP and ADP in lymphocytes from rats with induced sepsis.

## 2. Materials and Methods

### 2.1. Chemicals

Adenosine 5′-triphosphate disodium salt (ATP), adenosine 5′-diphosphate sodium salt (ADP), bovine serum albumine, Trizma base, Trypan Blue solution, and Coomassie Brilliant Blue G were obtained from Sigma-Aldrich (St. Louis, MO, USA). Ficoll-Hypaque (Lymphoprep) was purchased from Nycomed Pharma (Oslo, Norway). Physiological solution (0.9 g NaCl/100 mL distilled water) was obtained from Fresenius KABI (Brazil). K_2_HPO_4_ was purchased from Reagen (Brazil). All chemicals used in the experiments were of analytical grade and of the highest purity.

### 2.2. Animals

Male and female Wistar rats of 200–300 g bodyweight were used for all experiments, which were performed in accordance with the guidelines of Committee on Care and Use of Experimental Animal Resources (UFSM, Brazil) and in accordance with international guidelines.

### 2.3. Sepsis Induction and Samples Preparation

Animals were randomly divided into two groups (5 rats in each group): control and induced sepsis. To induce sepsis, it was used a model of cecal ligation and puncture as previously described [12]. After 12 h of induction, animals were anesthetized with isoflurane and the whole blood was collected through cardiac puncture in tubes containing ethylenediamine tetraacetic acid.

### 2.4. Isolation of Mononuclear Cells from Blood

Lymphocyte-rich mononuclear cells were isolated from blood collected with ethylenediamine tetraacetic acid and separated on Ficoll-Histopaque density [13] as previously described. The percentage of lymphocytes was superior to 93% as previously described [14].

### 2.5. NTPDase Activity Assay

After the isolation of lymphocytes, the NTPDase activity was determined as described previously by our group [15], measuring the amount of liberated inorganic phosphate (Pi) using a colorimetric assay. The reaction medium contained 0.5 mM CaCl_2_, 120 mM NaCl, 5 mM KCl, 60 mM glucose, and 50 mM Tris-HCl buffer (pH 8.0) in a final volume of 200 *μ*L. Then 20 *μ*L of intact mononuclear cells suspended in saline solution was added to the reaction medium (2–4 *μ*L protein) and preincubated for 10 min at 37°C. The reaction was started by adding the substrate (ATP or ADP) at a final concentration of 2 mM and was stopped with 200 *μ*L of 10% trichloroacetic acid to provide a final concentration of 5%. The samples were chilled on ice for 10 min before assaying the release of Pi as described previously [16], using malachite green as a colorimetric reagent and KH_2_PO_4_ as a standard. Light absorbance was measured at 630 nm in a spectrophotometer (Biospectro SP-22). Control reactions were performed by adding the enzyme preparation after the addition of trichloroacetic acid to correct for nonenzymatic nucleotide hydrolysis. All samples were run in triplicate, and the specific activity is reported as nanomoles of Pi released per minute per milligram of protein.

### 2.6. Protein Determination

Protein was measured by the Coomassie blue method as described previously [17]. Briefly, a solution of Coomassie (117 *μ*L Coomassie, 0.85 M ethyl alcohol, and 1.46 M ortho-phosphoric acid) was prepared. A standard curve with bovine serum albumin varying from 0.1 to 0.5 mg of protein per milliliter was performed. To quantify the protein content, 50 *μ*L of sample was added to 2.5 mL of Coomassie solution and, after 5 min, the absorbance was read in a spectrophotometer (Biospectro SP-22) at 595 nm.

### 2.7. Anatomopathologic Analysis

Samples of liver, kidney, and lung tissue, *ex vivo*, were collected and fixed in 10% formalin solution and then dehydrated and embedded in paraffin, followed by sectioning and histological staining with hematoxylin and eosin (H&E). The slides were observed in an optical microscope (400x) to check for possible changes in the respective tissues.

### 2.8. Statistical Analysis

Statistical analysis was performed using the nonparametric Mann-Whitney test, since results did not show Gaussian distributions. *P* < 0.05 was considered to represent a significant difference among the analyses performed. All data were expressed as mean ± standard error of the mean.

## 3. Results

### 3.1. Clinical Features

After 12 h of sepsis induction, the animals showed swollen abdominal region with fecal material release, as described by Benjamin [18].

### 3.2. NTPDase Activity in Lymphocytes

**Figure 1:**
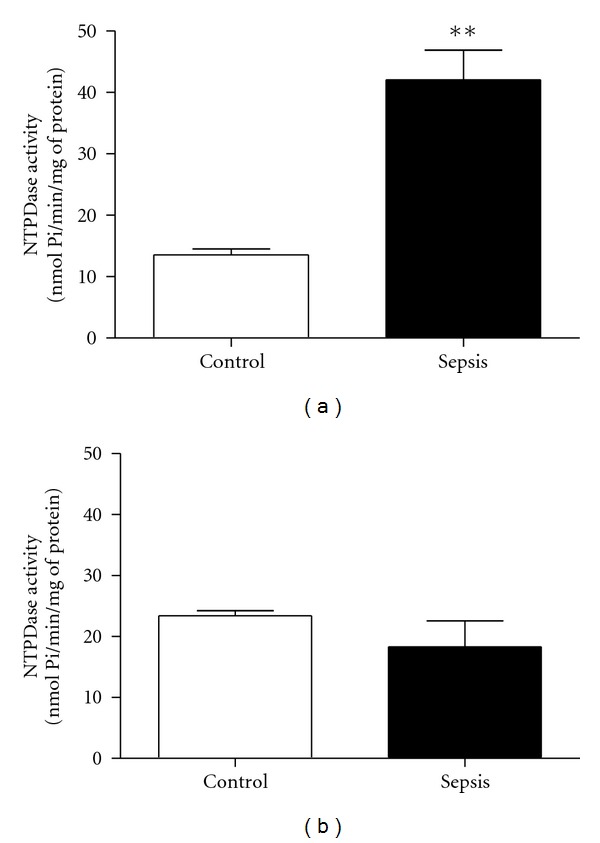
NTPDase activity in peripheral blood lymphocytes from rats with induced sepsis using (a) ATP or (b) ADP as a substrate. Bars represent means ± standard error of the mean (*P* < 0.01, *n* = 5). Mann-Whitney test. **Indicates a significant difference compared with control.

The statistical analysis demonstrated that animals from septic group had an increased ATP hydrolysis (*P* < 0.01) (Figure 1(a)) but did not show any statistical difference (*P* > 0.05) in regard to ADP hydrolysis (Figure 1(b)).

### 3.3. Anatomopathologic Analysis

**Figure 2:**
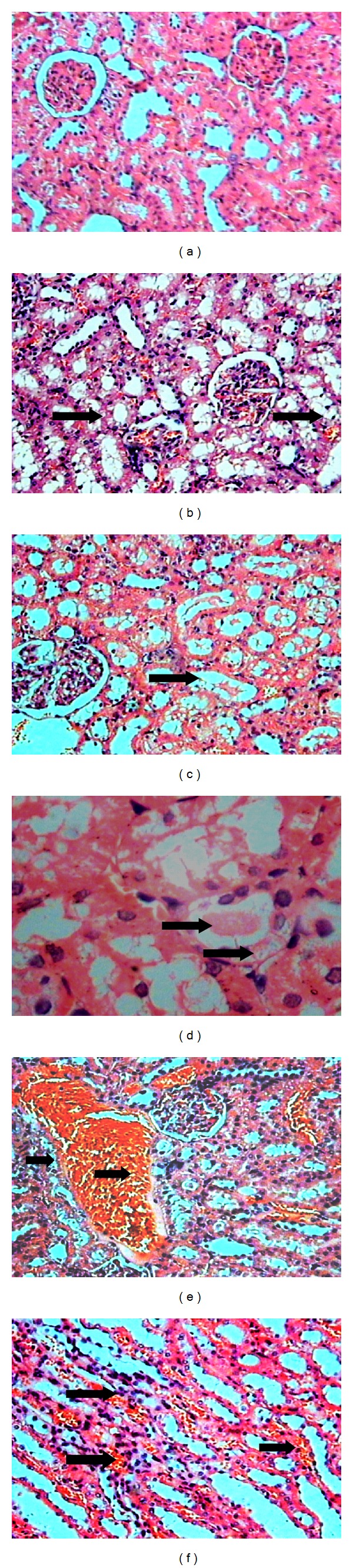
(a) Normal architecture of kidney with normal convoluted tubules and glomeruli (HE, 10x). (b) Abnormal kidney of rat with induced sepsis showed architecture with cellular vacuolization (degeneration) (HE, 10x). (c) Congested tubules (HE, 10x). (d) Congested convoluted proximal tubules lined by vacuolar cells with absence of individual tubular cells (HE, 40x). (e) Presence of substantial vascular congestion in capllaries in the cortex and desquamation of cell fragments into lumen (HE, 10x). (f) The medullar area shows congested peritubular capillaries (vasa recta) with discrete polymorphonuclear infiltrate (HE, 10x).

Kidney section of control group showed normal (A) glomeruli and convoluted tubules within renal cortex. No abnormal proliferations were seen. The medulla and hilum were microscopically normal. Atypical congested convoluted tubules were seen in kidneys of rats with induced sepsis. In some areas, the convoluted tubules were lined by crowded hyperchromatic cuboidal cells, which had decreased cytoplasm. Kidney sections also showed some congested glomeruli, besides tubular necrosis with multiple points along the nephron with cellular vacuolization (degeneration) and necrosis. Shedding or desquamation of cell fragments into lumen and absence of individual tubular cells were also viewed. Presence of marked vascular congestion in arterioles and peritubular capillaries in the renal cortex and medullar area were observed (Figure 2). These findings suggest tubular necrosis.

**Figure 3:**
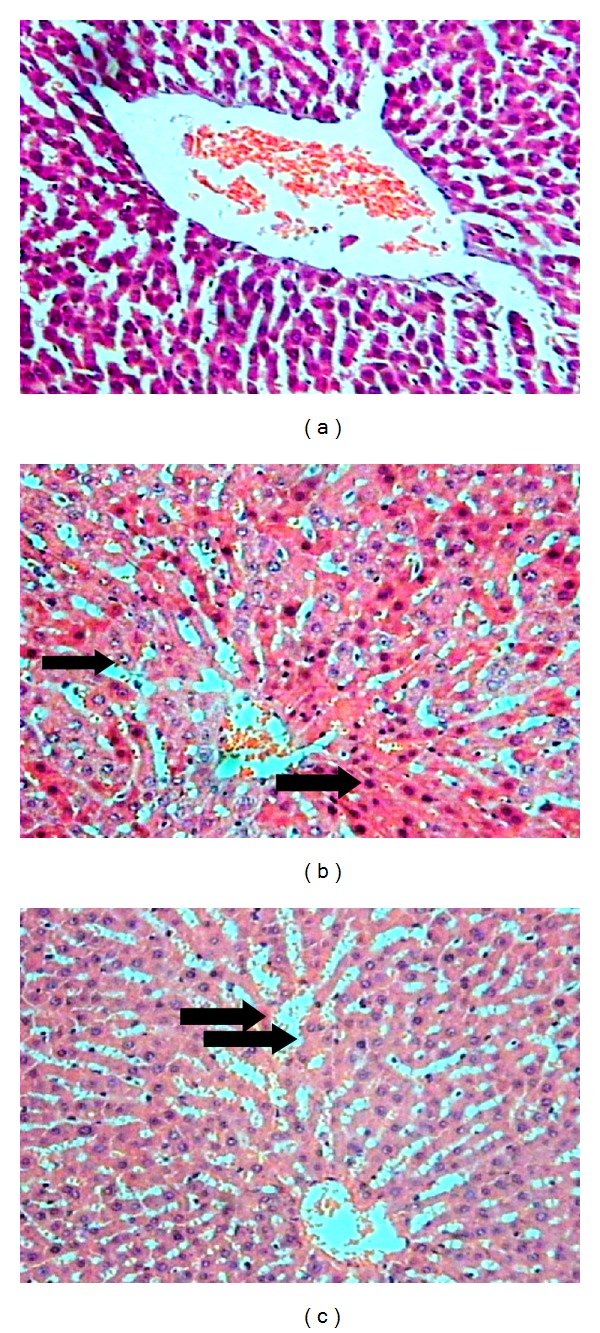
(a) Liver sections of control rats showing normal histological appearance (HE, 10x). (b) Liver of the rats with induced sepsis showed a partial hepatic cord disarrangement and an increased number of hepatocytes in process of involution with pyknotic nuclei and condensed cytoplasm (HE, 10x). (c) The central vascular sinusoids are dilated and congested filled with erythrocytes. Periportal hepatocytes are normal (*not showed*) (HE, 10x).

The liver section of control group showed normal histological appearance with normal polyhedral hepatocytes. The rats with induced sepsis showed important morphologic changes, as vascular congestion and some marks of necrosis.  The microscopic analysis showed partial hepatic cord disarrangement and increased number of hepatocytes undergoing involution with pyknotic nuclei and condensed cytoplasm. The central veins, spaces of Disse, and central vascular sinusoids were dilated and congested, filled with erythrocytes (Figure 3).

**Figure 4:**
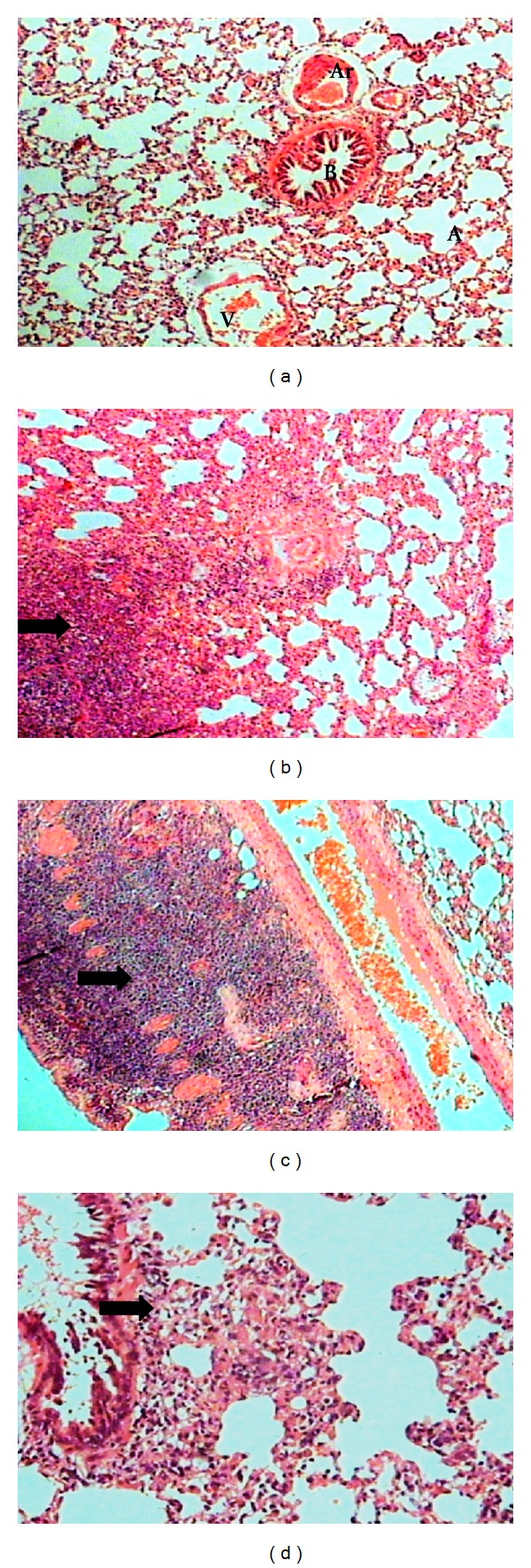
(a) Lung section of control group with normal histological appearance of alveolus (A), bronchiole (B), arteriole (Ar) and venule (*V*) (HE, 10X). (b) Lung of rats with induced sepsis showing different degrees of lung consolidation and alveolar spaces were infiltrated with a large number of mononuclear cells (HE, 10x). (c) Presence of mononuclear infiltrated in the wall of bronchiole (HE, 10x). (d) Local thickening of infiltrated mononuclear cells in alveolar wall and peribronchial area (HE, 20x).

The lung section of control group showed normal histological appearance showing a bronchiole and adjacent alveoli, airways, blood vessel, and parenchyma with delicate alveolar septal tissues. The rats with induced sepsis different degrees of lung consolidation developed and alveolar spaces were infiltrated with a large number of mononuclear cells (Figure 4).

## 4. Discussion

The extracellular nucleotides are important signaling molecules, being essential to start and maintain the inflammatory reactions [19]. These nucleotides are found in high concentrations within cells when compared to the extracellular environment, which is a characteristic of signaling molecules. Thus, in response to different stimulus or conditions, including damage to plasmatic membrane induced by hypoxia, ischemia, or inflammation, increasing concentrations of nucleotides can be released to the extracellular environment [20]. During the inflammatory process, ATP is involved in the development of inflammation by several processes: released histamine from mastocytes, production of prostaglandins, and production and release of cytokines from immune cells [21]. Besides these release forms, related mainly to cellular injury, ATP may be released from intact cells by physiological mechanisms, as it occurs, for example, in nervous transmission.

In this study, histological analysis showed several morphological changes in organs analyzed from septic group in tissues such as kidney, liver, and lung. Among the more outstanding changes include the marked vascular congestion, necrosis, and infiltration of mononuclear cells in agreement with previous studies [22] in lung tissue.

Furthermore, the results also demonstrated that the animals induced to sepsis had an increase in the NTPDase activity (ATP as a substrate) and no alterations in the ADP hydrolysis. The increased NTPDase activity in lymphocytes and the morphological alterations observed in the septic group are probably related with a physiological increase of ATP in the extracellular milieu. A physiological role for ATP is the regulation of inflammation [23], and it is known that once released, it interacts with specific receptors, denominated purinergic receptors, which establish a communication between cells [24]. The action of ATP during the inflammatory process occurs primarily via activation of purinergic P2X receptors, which may lead the cellular apoptosis [25]. Extracellular ATP has been shown to induce shedding of L-selectin from T lymphocytes via activation of purinergic P2X7 receptors [26–30]. Since L-selectin is primarily involved in lymphocyte homing to lymphoid tissues and is shed upon lymphocyte activation, ATP was proposed to be involved in migration of activated lymphocytes to sites of inflammation [31]. Additionally, there was verified [32] a possible involvement of P2X7 receptors in the lymphocytes proliferation and the activation of interleukin-2 transcription factor as a consequence. Interleukin-2 is a proinflammatory cytokine as interleukin-6, tumor necrosis factor-*α*, and interferon-*γ* [1], which participate in the inflammatory response regulation in sepsis [33]. These results corroborate with our findings, which suggest an increased extracellular ATP level in the animals induced to sepsis.

It is proposed that the systemic injuries promoted by this induced clinical condition may have increased the extracellular levels of ATP as a consequence of the cellular damage observed. In this context, the increased ATPasic activity was probably induced as a dynamic response to clean up the elevated ATP levels resulting from cellular death.

##  Conflict of Interests

There is no actual or potential conflict of interest.
